# Enzymatic vitamin A_2_ production enables red-shifted optogenetics

**DOI:** 10.1007/s00424-023-02880-2

**Published:** 2023-11-21

**Authors:** Johanna Gerhards, Leo I. Volkov, Joseph C. Corbo, Daniela Malan, Philipp Sasse

**Affiliations:** 1 Institute of Physiology I, Medical Faculty, University of Bonn, 53125 Bonn, Germany; 2 Department of Pathology and Immunology, Washington University School of Medicine, St. Louis, MO 63110 USA

**Keywords:** Optogenetics, ChR2, ReaChR, Vitamin A_2_, Cyp27c1

## Abstract

Optogenetics is a technology using light-sensitive proteins to control signaling pathways and physiological processes in cells and organs and has been applied in neuroscience, cardiovascular sciences, and many other research fields. Most commonly used optogenetic actuators are sensitive to blue and green light, but red-light activation would allow better tissue penetration and less phototoxicity. Cyp27c1 is a recently deorphanized cytochrome P450 enzyme that converts vitamin A_1_ to vitamin A_2_, thereby red-shifting the spectral sensitivity of visual pigments and enabling near-infrared vision in some aquatic species.

Here, we investigated the ability of Cyp27c1-generated vitamin A_2_ to induce a shift in spectral sensitivity of the light-gated ion channel Channelrhodopsin-2 (ChR2) and its red-shifted homolog ReaChR. We used patch clamp to measure photocurrents at specific wavelengths in HEK 293 cells expressing ChR2 or ReaChR. Vitamin A_2_ incubation red-shifted the wavelength for half-maximal currents (λ_50%_) by 6.8 nm for ChR2 and 12.4 nm for ReaChR. Overexpression of Cyp27c1 in HEK 293 cells showed mitochondrial localization, and HPLC analysis showed conversion of vitamin A_1_ to vitamin A_2_. Notably, the λ_50%_ of ChR2 photocurrents was red-shifted by 10.5 nm, and normalized photocurrents at 550 nm were about twofold larger with Cyp27c1 expression. Similarly, Cyp27c1 shifted the λ_50%_ of ReaChR photocurrents by 14.3 nm and increased normalized photocurrents at 650 nm almost threefold.

Since vitamin A_2_ incubation is not a realistic option for *in vivo* applications and expression of Cyp27c1 leads to a greater red-shift in spectral sensitivity, we propose co-expression of this enzyme as a novel strategy for red-shifted optogenetics.

## Introduction

Optogenetics is a technique that involves the use of light to control the function of genetically modified cells or organs with light-sensitive proteins. In the last decade, optogenetics has become a powerful method for light-induced modulation of electrical excitability, calcium influx, or stimulation of intracellular signaling pathways with high temporal and spatial precision [9]. Several optogenetic actuators of the rhodopsin family containing light-gated ion channels or pumps have been described and used for various applications [3, 4, 9, 21]. Among these, ChR2, a light-induced cation channel, was used to depolarize cardiomyocytes in vitro and in the intact mouse heart, enabling pacing the whole heart [6]. Using stronger, spatially extended, and longer periods of illumination, ChR2 can also be used to defibrillate the heart and to terminate ventricular arrhythmia if sufficient transmural activation of ChR2 is achieved [5].

ChR2 and its homologs are maximally activated by light between 450 nm and 545 nm [20]*.* However, light in this range has limited organ penetration because it is strongly absorbed and scattered by tissue and blood [5, 17]. Since red wavelengths are less readily absorbed, opsins with spectral sensitivity in the red to near-infrared range (650 nm-900 nm) would enable better tissue penetrance [7, 17]. Several red-shifted channelrhodopsin variants have been developed by mutagenesis and screening, but most have less optimal properties such as lower expression levels and reduced photocurrents, or slower gating kinetics than the classical ChR2 [7, 18, 28]. One promising red-shifted homolog is ReaChR, with an activation maximum between 590 and 630 nm when measuring stationary plateau photocurrents [15, 19].

Another strategy for achieving red-shifted sensitivity of optogenetic actuators would be to exchange the chromophore of the optogenetic actuator [27, 28]. Vitamin A_1_-derived all-trans-retinal, the common chromophore in microbial opsins, can be replaced by vitamin A_2_-derived all-trans 3,4-didehydroretinal, which has a red-shifted absorption spectrum due to an extended system of conjugated double bonds [24, 27]. The use of this chromophore in photoreceptors in cold-blooded vertebrates enhances vision in turbid aquatic environments where long wavelength light predominates [14]. Application of high concentrations of exogenous all-trans 3,4-didehydroretinal to ChR2 was shown to result in a 30-40 nm red-shift of half-maximal currents [28]. Nevertheless, substitution of all-trans 3,4-didehydroretinal is not a convenient option for optogenetic stimulation in living organisms because endogenous vitamin A_1_ in the form of all-trans-retinol is the predominant form in the mammalian system [10, 22, 29, 31] and would outcompete exogenously supplied vitamin A_2_ [28].

Recently, the enzyme responsible for converting vitamin A_1_ to A_2_ in the visual pigments of some fish and amphibians was discovered to be Cyp27c1, a dehydrogenase belonging to the Cytochrome P450 family [10]. Here, we show that the co-expression of Cyp27c1 with an optogenetic actuator provides a novel strategy to achieve red-shifted optogenetics. We expressed Cyp27c1 together with ChR2 as well as with the red-shifted homolog ReaChR in HEK 293 cells and analyzed the spectral sensitivity of photocurrents. We showed that Cyp27c1 overexpression in HEK cells results in the conversion of vitamin A_1_ to A_2_, leading to a red-shift in the spectral sensitivity of both ChR2 and ReaChR channelrhodopsins.

## Methods

### Generation of the CAG CYP27c1 EGFP Vector

To stably express zebrafish (*Danio rerio*) Cyp27c1 we engineered an expression vector by digesting the AAV_pVDM2_Dr_Cyp vector [10] containing Cyp27c1 and EGFP linked by a T2A self-cleaving peptide with BlpI and BsrGI. The resulting 3009 bp fragment was subcloned into the 5895 bp fragment of the pCAG hChR2-EYFP vector [6] digested with the same enzymes and containing a neomycin selection cassette. The resulting vector CAG-Cyp27c1-EGFP was verified by restriction enzyme analysis and sequencing. The pEGFP-N1_ReachR_mCerulean vector was received from J. Vierock for expression of ReaChR mCerulean under the control of a CMV promoter.

### HEK 293 cell transfection and culture

HEK 293 cells (AD-293, ATTC) were maintained in Dulbecco’s Modified Eagle’s Medium (DMEM, Life Technologies) supplemented with 10% FCS (Capricorn Scientific), 0.1 mM nonessential amino acids, 100 U/ml penicillin, 100 mg/ml streptomycin and 0.1 mM β-mercaptoethanol (all supplements Thermofisher), plated onto fibronectin coated coverslips (20.000 cells on 22 mm^2^ glass coverslips) and grown for 24 h. Transfection was performed at 60-80% confluence with Fugene HD (Promega) using 2-3 μg DNA per well. To obtain a stable Cyp27c1 cell line, CAG-Cyp27c1-EGFP was linearized with PvuI before transfection and cells were selected with G418 (Thermofisher, 600 μg/ml). ChR2 or ReaChR was transfected transiently and cells were investigated after 48-72 h of culture in FCS-free Panserin 293A medium (Pan Biotech) in which vitamin A_1_ (as retinol-acetate) is present at 0.4 μM as stated by the supplier. For some patch clamp and HPLC experiments the medium was supplemented with vitamin A_1_ (all-trans-retinol, R7632, Sigma) or vitamin A_2_ (all-trans-3,4-didehydroretinol, sc-209587, Santa Cruz Biotechnology) for at least 24 h.

### Patch clamp analysis of photocurrents

Patch clamp recordings were performed using an EPC10 amplifier (Heka) at 10 kHz sampling rate on a Nikon Ti2 microscope with 20x objective (S Fluor DIC M/N2 Nikon) at 35 ± 2°C with an external solution of (in mM) 140 NaCl, 5.4 KCl, 1.8 CaCl_2_, 2 MgCl_2_, 10 glucose, 10 Hepes (pH 7.4, NaOH), and an internal solution of 50 KCl, 80 K-aspartate, 1 MgCl_2_, 3 MgATP, 10 EGTA, 10 HEPES (pH 7.2, KOH). Pipettes were pulled from borosilicate glass (BF 150-86-10, Scientific Products) at a tip resistance of 4-6 MΩ using a P-1000 puller (Sutter Instrument). Only ChR2-EYFP or ReaChR-Cerulean positive cells were investigated. Light was generated at 10 nm wavelength steps with a Xenon arc lamp-based monochromator (Optoscan, Cairn) controlled by the patch clamp amplifier and a custom-coded microcontroller (Arduino) to obtain a uniform slit bandwidth of 30 nm. The activation spectrum was analyzed from 480 nm to 600 nm for ChR2 nm and from 590 nm to 700 nm for ReaChR. Light intensities were calibrated at the objective plane using a power meter (PM100A with S170C sensor, Thorlabs) and reported in Table 1.

**Table 1:** Mean light intensities at indicated wavelenghts obtained from the monochromator at the objective plane

ChR2 (nm)	480	490	500	510	520	530	540	550	560	570	580	590	600
mW/mm^2^	7.2	6.7	6.4	6.1	5.9	5.6	5.4	5.1	4.8	4.6	4.3	4.0	3.7
ReaChR (nm)	590	600	610	620	630	640	650	660	670	680	690	700	
mW/mm^2^	3.7	3.4	3.2	2.9	2.7	2.6	2.6	2.7	2.7	2.6	2.4	2.1	

Photocurrents were evoked every 6 s at a holding potential of -40 mV and steady-state currents were measured at the end of a 500 ms light pulse (300-495 ms after the start of illumination) subtracted by baseline currents (250-475 ms after end of illumination). Photocurrents were normalized to the cell capacity. Because we measured saturating photocurrents (see also discussion), photocurrents were not normalized to differences in light intensity. Photocurrents are presented as absolute currents, or to account for cell-to-cell variability in expression, for spectral comparisons photocurrents were normalized to the maximal value of each cell.

### Immunohistochemistry

Cells were fixed in 4% PFA and stained with anti-Cyp27c1 ([10], 1:300) or mitochondrial ATP synthase F1-β antibody (provided by W. Voos, 1:80) for 2 h. Alexa-Fluor goat anti-rabbit 647 or Alexa Fluor goat anti-mouse 555 (Life Technologies) secondary antibodies (1:400) were then applied for 1 h in a solution with 1 μg/ml Hoechst 33342 (Sigma-Aldrich) as a nuclear counterstain. Immunostainings were imaged using a Zeiss LSM confocal or a Nikon Eclipse Ti2 microscope.

### High performance liquid chromatography (HPLC)

HEK 293 cells incubated in vitamin A_1_ (all-trans-retinol) were detached, centrifuged, frozen on dry ice and stored at -80^o^C. Samples were subsequently thawed and homogenized in cold 0.9% NaCl solution by sonication. A small portion of the homogenized sample was used to measure protein content for normalization. Retinaldehydes were derivatized and analyzed by HPLC as previously described [30]. Retinoids were identified by comparison to retinol and 3,4-didehydroretinol standards as well as the published literature [1, 12, 23]. Standard curves were generated using retinol (R7632, Sigma) for the quantification of A_1_ retinols (all-trans-retinol and 13-cis-retinol) and 3,4-didehydroretinol (sc-209587, Santa Cruz Biotechnology) for the quantification of A_2_ retinols (all-trans-3,4-didehydroretinol and 13-cis 3,4-didehydroretinol). Calculations were made based on previously published extinction coefficients [2]. Because incubation of cells with 3.5 and 35 μM vitamin A_1_ showed similar results, we pooled these data for statistical analysis of the Cyp27c1 effect on vitamin A_2_ production.

### Statistical analysis

Data were analyzed offline using Fitmaster (Heka), Graph Pad Prism 6 (GraphPad) and Origin (OriginLab) software. Data are shown as mean ± SEM. Statistical analysis was performed with unpaired t-test or one-way ANOVA with Tukey’s multiple comparison test. A *p* value ≤ 0.05 was considered statistically significant.

## Results

### Vitamin A_2_ incubation causes a red-shift in spectral sensitivity

**Fig. 1:**
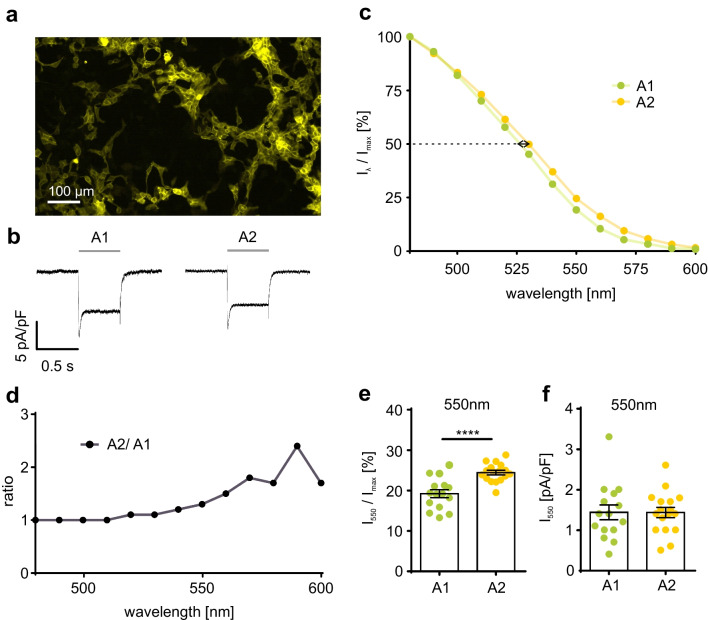
Spectral sensitivity of ChR2. **a** Expression of ChR2 in fusion to EYFP (yellow) in HEK 293. **b** Representative ChR2 currents evoked by illumination (480 nm, 0.5 s, grey line) in ChR2 expressing HEK 293 cells in Panserin 293A medium supplemented with 25 μM vitamin A_1_ (A1) or vitamin A_2_ (A2) 24-48 h before experiments. **c** ChR2 steady-state photocurrent at different wavelengths in relation to the maximal current for each cell in the presence of vitamin A_1_ (25 μM, n = 15) or A_2_ (25 μM, n = 17). Spectral difference at 50% photocurrent highlighted by black arrow. **d** Ratio of normalized ChR2 currents in the presence of vitamin A_2_ and A_1_. **e,f** Quantification of normalized (**e**) and absolute (**f**) ChR2 photocurrents at 550 nm in the presence of vitamin A_1_ and A_2_. **** p ≤ 0.0001

In order to analyze the effect of exogenously supplied vitamin A_1_ or vitamin A_2_ on the spectral sensitivity of ChR2, we incubated HEK 293 cells expressing ChR2 in fusion with EYFP (Fig. 1a) with vitamin A_1_ (A1, 25 μM) or vitamin A_2_ (A2, 25 μM) and measured photocurrents in response to exposure to light of varying wavelengths. We supplied vitamin A_1_ and A_2_ as the retinol form to mimic the in vivo situation where the majority of circulating vitamin A is in the alcohol form. We assume that the alcohol form is converted by cells at low rates into the aldehyde form by endogenous dehydrogenases and then taken up by the optogenetic actuators (see Discussion). The response to 480 nm light is shown in Fig. 1b. To precisely control the concentration of vitamin A_1_ or A_2_ in the medium, we used the serum-free medium Panserin 293A, which contains 0.4 μM vitamin A_1_ (as all-trans-retinol) because standard FCS contains undefined quantities of vitamin A. As shown previously [28], vitamin A_2_ incubation led to a red-shift of the spectral sensitivity of ChR2 (Fig. 1c, arrow) and increased photocurrents at wavelengths between 525 nm and 600 nm (Fig. 1d). The wavelength at which half-maximal currents are obtained (λ_50%_) was red-shifted by 6.8 nm after vitamin A_2_ incubation. At 550 nm, where the effect can be clearly seen, cells incubated with vitamin A_2_ reached 24.5 ± 0.5%, whereas only 19.2 ± 1.0% of the maximal photocurrent was measured in cells incubated with vitamin A_1_ (Fig. 1e). Absolute photocurrents were not different (Fig. 1f) most likely because maximal photocurrents were larger in vitamin A_1_.than in vitamin A_2_, which was discussed before [8].

**Fig. 2:**
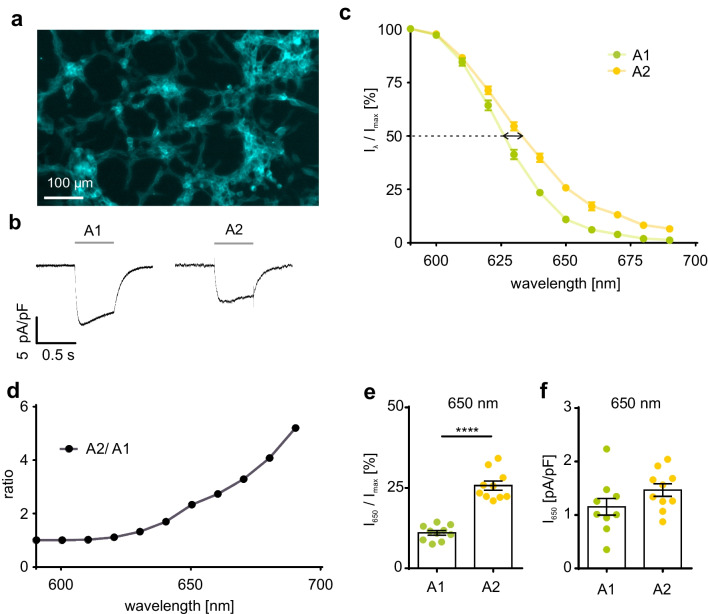
Spectral sensitivity of ReaChR. **a** Expression of ReaChR in fusion to Cerulean (blue) in HEK 293 cells. **b** Representative ReaChR currents evoked by illumination (600 nm, 0.5 s, grey line) in ReaChR expressing HEK 293 cells in Panserin 293A medium supplemented with 25 μM vitamin A_1_ (A1) or vitamin A_2_ (A2) 24-48 h before experiments. **c** ReaChR steady-state photocurrent at different wavelengths in relation to the maximal current for each cell in the presence of vitamin A_1_ (25 μM, n = 10) or A_2_ (25 μM, n = 10). Spectral difference at 50% photocurrent highlighted by black arrow. **d** Ratio of normalized ReaChR currents in the presence of vitamin A_2_ and A_1_. **e,f** Quantification of normalized (**e**) and absolute (**f**) ReaChR photocurrents at 650 nm in the presence of vitamin A_1_ and A_2_. **** p ≤ 0.0001

Next, we investigated the effect of vitamin A_2_ on the red-shifted channelrhodopsin variant ReaChR. Consistent with our ChR2 experiments (Fig. 1), HEK 293 cells with stable expression of ReaChR in fusion to Cerulean (Fig. 2a, b) showed a significant red-shift of 12.4 nm of λ_50%_ after incubation with vitamin A_2_ (Fig. 2c). ReaChR-expressing cells incubated with vitamin A_2_ showed ~2.5-fold higher photocurrents at the long wavelength edge (~660 nm) of spectral sensitivity than those incubated with vitamin A_1_ (Fig. 2d). For instance, cells incubated with vitamin A_1_ achieved 11.0 ± 0.7% at 650 nm, whereas cells incubated with vitamin A_2_ attained 25.7 ± 1.4% of their maximal photocurrent (Fig. 2e). Absolute photocurrents were not different (Fig. 2f) most likely because maximal photocurrents were larger in vitamin A_1_. Having shown that vitamin A_2_ is able to red-shift spectral sensitivity of ChR2 and ReaChR, we expect similar effects of vitamin A_2_ application in other channelrhodopsin variants.

### Cyp27c1 converts vitamin A_1_ to vitamin A_2_

**Fig. 3:**
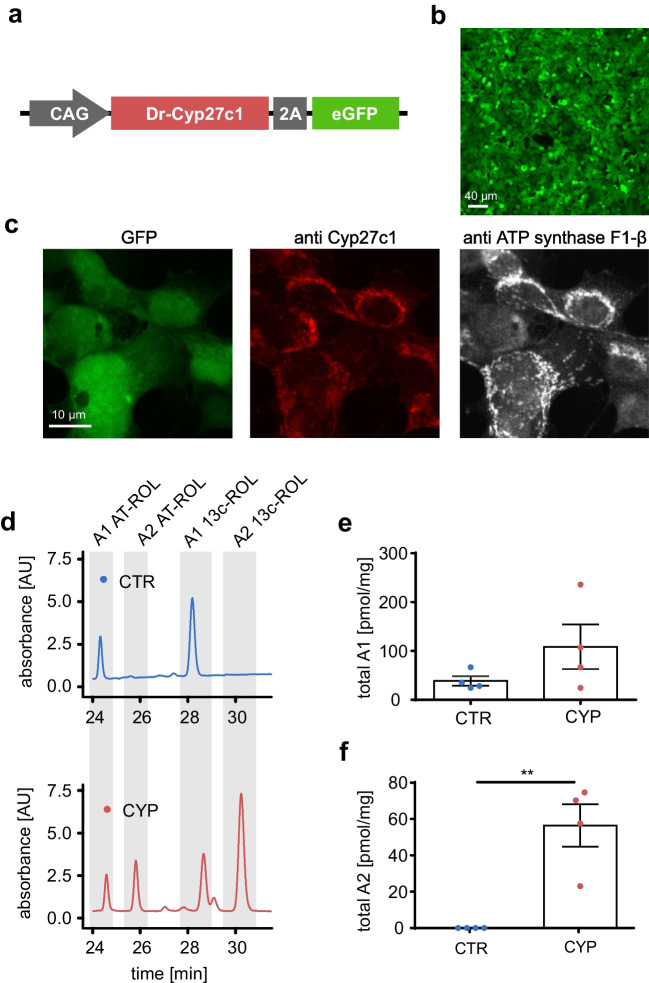
Cyp27c1 expression converts vitamin A_1_ into vitamin A_2_**.** **a** Plasmid for expression of zebrafish Cyp27c1 and EGFP separated by a 2A self-cleaving peptide. **b,c** HEK 293 cells stably expressing Cyp17c1-EGFP (green) in overview (**b**) and stained with antibodies (**c**) against Cyp27c1 (red) and mitochondrial ATP synthase F1-β (white). **d** HPLC analysis of HEK 293 cells without (CTR) and with Cyp27c1 (CYP) expression after incubation with 3.5 μM vitamin A_1_ (all-trans retinol). Specific peaks for all-trans retinol (AT-ROL) and 13-cis retinol (13c-ROL) species of vitamin A_1_ and A_2_ highlighted in grey. Note the appearance of vitamin A_2_ species only in Cyp27c1 expressing cells. **e,f** Quantification of total amount of vitamin A_1_ (**e**, n = 4) or vitamin A_2_ (**f**, n = 4) normalized to total protein level in HEK 293 cells without (CTR) and with Cyp27c1 (CYP) expression after incubation with vitamin A_1_ (pooled data for 3.5 and 35 μM vitamin A_1_). ** p ≤ 0.01

For *in vivo* optogenetic applications, the use of exogenously applied vitamin A_2_ as a chromophore may not be feasible due to the predominance of vitamin A_1_ in mammalian serum and tissues [10, 22, 29, 31]. In order to take advantage of the red-shifting effect of vitamin A_2_ in the presence of vitamin A_1_, we used the vitamin A_1_-to-A_2_ converting enzyme Cyp27c1 [10]. We generated a HEK 293 cell line stably expressing Cyp27c1 under the control of the CAG promoter (Fig. 3a) using neomycin selection (Fig. 3b). Comparison of EGFP-fluorescence signals in Cyp27c1-expressing cells (Fig. 3c, green) with anti-Cyp27c1 (Fig. 3c, red) and mitochondrial staining (Fig. 3c, white) suggests mitochondrial localization of Cyp27c1. To confirm the functionality of Cyp27c1 in HEK 293 cells, we performed HPLC analysis in lysates of HEK 293 cells expressing Cyp27c1 and wild-type controls and incubated them with 3.5 or 35 μM vitamin A_1_ for 24 h. HPLC analysis showed the presence of both vitamin A_1_ and A_2_ in cells expressing Cyp27c1 but only vitamin A_1_ in control cells (Fig. 3, d-f).

### Cyp27c1 red-shifts the spectral sensitivity of ChR2

**Fig. 4:**
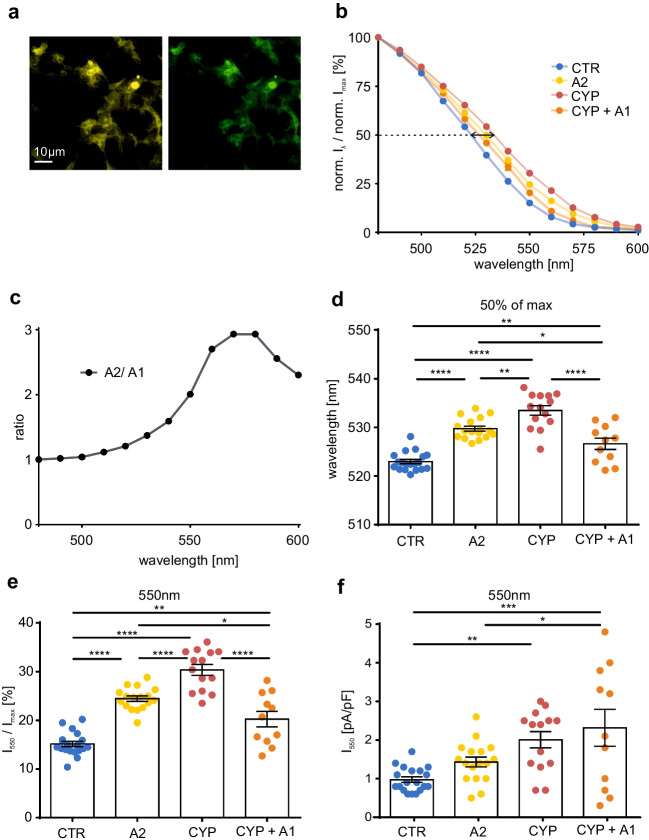
Cyp27c1 co-expression red-shifts the spectral sensitivity of ChR2 **a** Transient expression of ChR2-EYFP (yellow) in a HEK 293 cell line stably expressing Cyp27c1-EGFP (green). Special fluorescence filters were chosen to discriminate between EYFP and EGFP. **b** ChR2 photocurrents in relation to the maximal current for each cell in Panserin 293A medium for cells expressing Cyp27c1 without (CYP, n = 14) and with vitamin A_1_ incubation (CYP + A1, 5 μM, n = 11) and in control cells without (CTR, n = 19) and with vitamin A_2_ incubation (A2, 25 μM, n = 17). Note that Cyp27c1 expression shifts the spectral sensitivity of ChR2 at 50% photocurrent (black arrow). **c** Ratio of normalized ChR2 photocurrents in cells expressing Cyp27c1 and control cells shows up to three times higher currents at wavelengths between 550 and 600 nm. **d** Wavelengths resulting in 50% of ChR2 photocurrents from data shown in **b** calculated for each cell by linear fitting. **e,f** Quantification of normalized (**e**) and absolute (**f**) ChR2 photocurrents at 550 nm.. * p ≤ 0.05, ** p ≤ 0.01, **** p ≤ 0.0001

To analyze the effect of Cyp27c1 on the spectral sensitivity of the most commonly used optogenetic actuator, we transiently expressed ChR2-EYFP in Cyp27c1-expressing (Fig. 4a) and wild-type control HEK 293 cell lines. Analysis of photocurrents at different wavelengths showed that co-expression of Cyp27c1 leads to a red-shift of the spectral sensitivity (Fig. 4b, arrow) which was even larger than that observed after incubation with vitamin A_2_ (Fig. 1c). Interestingly, the red-shifting effect of Cyp27c1 could be reversed by adding 5 μM vitamin A_1_ to the culture medium, suggesting that the higher concentration of vitamin A_1_ led to replacement of the vitamin A_2_ chromophore (Fig. 4b). Photocurrents in Cyp27c1-expressing cells were up to three times higher at the long wavelength end of spectral sensitivity than control cells (Fig. 4c). Analysis of λ_50%_ for each cell individually showed a shift of 10.5 nm to longer wavelengths by Cyp27c1 expression (Fig. 4d), which underscores the potential benefits of Cyp27c1 co-expression for optogenetic applications. For example, at 550 nm, cells without Cyp27c1 expression reached only 15.2 ± 0.5% of their maximal currents (Fig. 4e), while Cyp27c1 expressing cells showed almost one-third of maximal currents (Fig. 4e, 30.4 ± 1.1%). Similarly, absolute photocurrents increased from 1.0 ± 0.1 pA/pF to 2.0 ± 0.2 pA/pF by Cyp27c1 expression Fig. 4f).

### Co-expression of Cyp27c1 red-shifts the spectral sensitivity of ReaChR

**Fig. 5:**
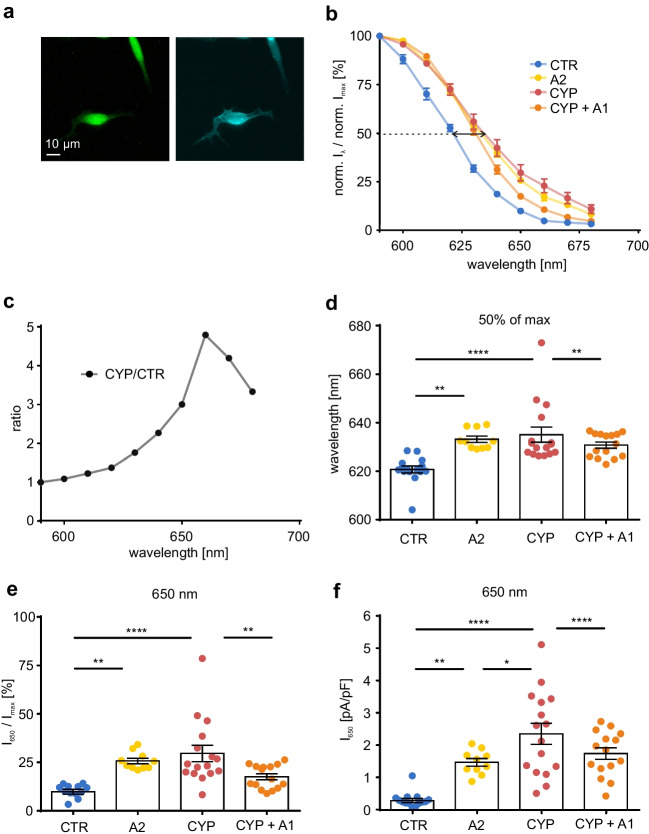
Cyp27c1 co-expression red-shifts spectral sensitivity of ReaChR **a** Transient expression of ReaChR-Cerulean (blue) in a HEK 293 cell line stably expressing Cyp27c1-EGFP (green). **b** ReaChR photocurrents in relation to the maximal current for each cell in Panserin 293A medium for cells expressing Cyp27c1 without (CYP, n = 15) and with vitamin A_1_ incubation (CYP + A1, 5 μM, n = 14) and in control cells without (CTR, n = 15) and with vitamin A_2_ incubation (A2, 25 μM, n = 14). Note that Cyp27c1 expression shifts the spectral sensitivity of ReaChR at 50% photocurrent (black arrow). **c** Ratio of normalized ReaChR photocurrents in cells expressing Cyp27c1 and control cells shows more than three times higher currents at wavelengths above 650 nm. **d** Wavelengths resulting in 50% of normalized ReaChR photocurrents from data shown in **b** calculated for each cell by linear fitting **e,f** Quantification of normalized (**e**) and absolute (**f**) ReaChR photocurrents at 650 nm from data shown in **b**. * p ≤ 0.05, ** p ≤ 0.01, **** p ≤ 0.0001

Co-expression of red-shifted ChR2 variants with Cyp27c1 might further extend spectral sensitivity into the far-red range. To investigate this, we tested the ChR2 homolog ReaChR in combination with Cyp27c1. HEK 293 cells co-expressing ReaChR and Cyp27c1 (Fig. 5a) showed a significant red shift of spectral sensetivity compared to control cells without Cyp27c1 co-expression (Fig. 5b**,** arrow). Adding vitamin A_1_ (5 μM) reversed the effect, indicating replacement of vitamin A_2_-derived chromophore by A_1_. (Fig. 5b). Photocurrents of Cyp27c1-expressing cells were up to four times higher at wavelengths between 630 nm and 690 nm in comparison to cells without Cyp27c1 expression (Fig. 5c). For example, at 650 nm, control cells only achieved 9.9 ± 1.3% of the maximal currents whereas 29.6 ± 4.2% of maximal currents was seen in Cyp27c1 expressing cells (Fig. 5e). Similarly, absolute photocurrents increased from 0.3 ± 0.1 pA/pF to 2.3 ± 0.3 pA/pF by Cyp27c1 expression (Fig. 5f). Analysis of λ_50%_ for each cell individually showed 635.0 ± 3.1 nm for Cyp27c1 expressing cells and 620.7 ± 1.4 nm for control cells (Fig. 5d), which constitutes a Cyp27c1-induced red-shift of λ_50%_ of 14.3 nm.

## Discussion

In this study, we explored the idea of red-shifting the spectral sensitivity of optogenetic actuators by co-expression of a vitamin A_1_-to-A_2_ converting enzyme, Cyp27c1. Consistent with the findings of Sineshchekov et al. [28], we showed that substitution of vitamin A_1_ with vitamin A_2_ leads to a red-shift of the spectral sensitivity of optogenetic actuators in living cells. Cyp27c1 was recently discovered to be the enzyme responsible for an enhancing near-infrared visual sensitivity by converting vitamin A_1_ to vitamin A_2_ in the visual pigments of aquatic vertebrates [10]. Thus, we here propose enzymatic vitamin A_2_ production by Cyp27c1 as a new method to red-shift the spectral sensitivity of optogenetic actuators without the need for supplying exogenous vitamin A_2_. Since longer wavelength light is less scattered and absorbed by tissue [5, 17], optogenetic actuators with spectral sensitivity in the red to near-infrared therapeutic window (650 nm-900 nm) can facilitate actuator operability at greater tissue depth. Several red-shifted channelrhodopsin variants have been developed from different species and using mutagenesis, but most have less optimal properties than ChR2 with respect to expression, kinetics, or ion selectivity [7, 18, 28]. So far, there is no evidence that the vitamin A_2_ chromophore significantly alters fundamental functions of optogenetic actuators other than their spectral sensitivity [28], although maximum photosensitivity of A_2_-based visual pigments may be reduced compared to A_1_-based pigments [8]. Thus, intracellular vitamin A_2_ production by Cyp27c1 is a promising alternative strategy to red-shift the spectral sensitivity of optogenetic actuators.

Sineshchekov et al. [28] describe a red-shift in the spectral sensitivity of peak photocurrents by ~30 nm for the channelrhodopsin variants CrChR1, CrChR2, CaChR1 and MvChR1 in HEK 293 cells after incubation with vitamin A_2_ in aldehyde form. However, our experiments with ChR2 and ReaChR showed only a 6.8 nm, and 12.4 nm shift in plateau photocurrents using identical vitamin A_2_ concentrations (25 μM), but in the alcohol form. Both differences (peak vs plateau and retinol vs retinal) might have contributed to the less pronounced red-shift we observed. Importantly, we specifically investigated the effect of Cyp27c1 on plateau stationary photocurrents because many optogenetic applications, including defibrillation [5] or seizure control, require not a single light pulse with peak photocurrents but repetitive or constant light-induced depolarization [5], and in this situation, peak photocurrents are desensitized [19, 20].

In contrast to earlier work that supplemented the oxidized versions of vitamin A_1_ (all-trans-retinal) and vitamin A_2_ (all-trans 3,4-didehydroretinal, not commercially available) [27, 28] we have supplied the reduced form (all-trans-retinol) because it is the dominant form in serum and tissue [10, 22, 29, 31] and is only converted to retinal in the eye by enzymes of the visual cycle [13]. Also, Cyp27c1 most efficiently catalyzes the conversion of retinol [10]. Thus, our findings in cell culture using retinol can be better transferred to the in vivo situation. Furthermore, all-trans-retinol in Panserin 293A medium is sufficient to generate large photocurrents in HEK293 cells. This is most likely due to the interconversion of retinal and retinol forms by endogenous dehydrogenases [13] such as the retinol dehydrogenase (RDH) 14, which is highly expressed in HEK293 cells [25]. We were not able to detect all-trans-retinal in HEK293 cells fed with all-trans-retinol by HPLC analysis after oximes derivatization (data not shown). However, the presence of photocurrents proofs that these small undetectable amounts of retinal generated by RDHs are sufficient as chromophore for microbial opsins. This is not surprising because, in contrast to bleaching opsins, which require a constant supply of retinal aldehyde for recovery aver every photocycle, ChR2 and other microbial opsins do not dissociate from the retinal molecule during the photocycle.

Interestingly, Sineshchekov et al. showed that the shift in spectral sensitivity due to vitamin A_2_ incubation correlated with the vitamin A_2_/A_1_ ratio in the medium [28], indicating a competition and similar binding affinities of both vitamins in the binding pocket of optogenetic actuators. The hypothesis of a vitamin A_2_/A_1_ competition is supported by our findings that the Cyp27c1-induced red-shift of spectral sensitivity can be reversed by elevating the vitamin A_1_ concentration (Fig. 4b,d, Fig. 5b,d). Thus, exogenous vitamin A_2_ application will not be an option for *in vivo* optogenetic applications because vitamin A_1_ is the predominant form in the mammalian serum and tissues [10, 22, 29, 31] and would compete with vitamin A_2_ and thereby reduce the red-shifting effect. In addition, high concentrations of exogenous vitamin A_2_ could have unknown systemic effects on the organism because elevated levels of 3,4-didehydroretinoids have been documented in hyperkeratotic lesions and some skin neoplasms in humans [10]. Overexpression of Cyp27c1 should also be tolerated by the immune system as orthologues can be found in most mammalian genomes, including the human genome [10]. For instance, Johnson et al. [11] report localization of Cyp27c1 in the skin although the function of vitamin A_2_ remains unclear.

Our experiments showed that Cyp27c1 is able to convert 0.4 μM vitamin A_1_ present in Panserin 293A medium into vitamin A_2_, thereby red-shifting the spectral sensitivity of co-expressed ChR2 or ReaChR. This is in line with literature describing the catalytic efficiency of Cyp27c1 as one of the highest among animal cytochrome P450 family members [10] and a maximal catalytic efficiency at a vitamin A_1_ concentrations between 0.1 and 2 μM. Because the levels of vitamin A_1_ (all-trans-retinol) in human blood of 0.5–1.5 μM [22, 29, 31] are within this range, we conclude that Cyp27c1 overexpression should be effective for *in vivo* red-shifted optogenetics at physiological vitamin A_1_ serum concentrations. Furthermore, our results show a higher red-shifting effect by Cyp27c1 (ChR2: 10.5 nm and ReaChR: 14.3 nm) than by vitamin A_2_ incubation (6.8 nm and 12.4 nm). This could be explained by a local intracellular production of vitamin A_2_ close to the chromophore binding site at the time of opsin maturation when the chromophore is being incorporated.

The vitamin A_2_-induced red-shift has been shown for numerous opsins [27, 28], corroborating the wide spectrum of possible applications of Cyp27c1 with vitamin A-based optogenetic actuators other than those we have tested. Sineshchekov et al. [28] predict the highest benefit by vitamin A_2_ for rhodopsins with intrinsically long-wavelength absorption in which the additional red-shift by Cyp27c1 might significantly increase their efficiency and finally allow optogenetic activation in deep layers. We can support this notion because with ReaChR and Cyp27c1 we found half-maximal photocurrents at 635 nm, approaching the therapeutic window of 650–900 nm.

In this study, we used high light intensities from a monochromator to determine the maximum possible plateau photocurrent. This required a large bandwidth of 30 nm and resulted in non-uniform intensities across the spectrum (Table 1). ChR2 plateau currents are almost fully saturated at the light intensities we have used [20], and ReaChR plateau photocurrents are saturated at 610 nm using ~ 2 mW/mm^2^ [19]. However, ReaChR could be not saturated for longer wavelengths which in turn would allow using higher light intensities to obtain larger photocurrents.

Potential applications of optogenetic tools in whole organisms with therapeutic purposes could be, among others, terminating ventricular tachycardia [5], seizure control in epilepsy [16], or restoring vision in patients with photoreceptor degeneration [26]. All these could benefit from optogenetic proteins with spectral sensitivity in the red-light range, enabling better tissue penetration and the use of less energetic and therefore less damaging long-wavelength photons. Given the greater red-shift due to Cyp27c1 expression compared to vitamin A_2_ incubation, we propose enzymatic vitamin A_2_ production by Cyp27c1 in combination with long wavelength optogenetic actuators as a complementary strategy for red-shifted optogenetics.

## Data Availability

Original data will be provided upon personal and reasonable request to the corresponding authors.
